# Variants in the Imprinted *IGF2* Gene: A Review and Phasing of De Novo Variants Using Long‐Read Sequencing

**DOI:** 10.1111/cge.70239

**Published:** 2026-09-01

**Authors:** Trine Maxel Juul, Susanne Eriksen Boonen, Katja Venborg Pedersen, Caroline Hey Bækgaard, Martin Larsen, Maria Kibæk, Niels Illum, Malene Heideman, Christiane Nielsen, Emilie Boye Lester, Christina Fagerberg

**Affiliations:** ^1^ Department of Clinical Genetics Odense University Hospital Odense Denmark; ^2^ Department of Clinical Research University of Southern Denmark Odense Denmark; ^3^ Department of Clinical Genetics Sygehus Lillebælt, Vejle Hospital Vejle Denmark; ^4^ H. C. Andersen Children's Hospital Odense University Hospital Odense Denmark; ^5^ Department of Pediatrics The Hospital of Southern Jutland, Aabenraa Hospital Aabenraa Denmark; ^6^ Department of Regional Health Research University of Southern Denmark Odense Denmark

**Keywords:** *IGF2*, imprinting, long‐read sequencing, Silver–Russell syndrome

## Abstract

Pathogenic variants on the paternal allele of *IGF2* are linked to Silver–Russell syndrome (SRS). This report describes two unrelated individuals—a 5‐year‐old girl and an adult female—with de novo IGF2 missense variants, both diagnosed with SRS. While one exhibited normal development, the other had intellectual disability, highlighting phenotypic variability. A review of 20 individuals with IGF2 variants revealed that SRS features, as defined by the Netchine‐Harbison Clinical Scoring System, were most common. Additional recurrent traits included delayed speech and motor development, under‐masculinized male genitalia, hand/foot anomalies, and congenital heart defects. Growth faltering patterns varied, and intellectual disability was seen in some. We also demonstrated that long‐read sequencing can determine the allelic origin of de novo IGF2 variants using differentially methylated regions, eliminating the need for parental samples. This approach confirms long‐read sequencing as a powerful tool for identifying de novo variant origins in imprinted genes like *IGF2*.

## Graphical abstract

Pathogenic *IGF2* variants on the paternal allele can cause Silver–Russell syndrome, with highly variable growth and neurodevelopmental outcomes. Long‐read sequencing can determine the parental origin of de novo *IGF2* variants via methylation patterns without parental samples, improving diagnostics for imprinted genes.

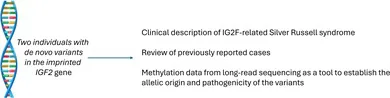

## 1 Introduction

Insulin‐like growth factor two (IGF‐II) is essential for regulating growth, especially in fetal life [1]. The imprinted IGF2 gene is predominantly expressed from the paternally inherited allele through epigenetic regulation by the H19/IGF2 imprinting control region (ICR1), which contains CTCF‐binding sites that regulate enhancer access and coordinates allele‐specific DNA methylation at secondary IGF2 differentially methylated regions (DMRs) and promoter usage [2, 3, 4]. While the imprinting‐associated methylation pattern persists after birth, IGF2 expression becomes tissue‐dependent because some postnatal transcripts arise from promoters that escape imprinting [5].

Pathogenic variants in *IGF2* are described to be associated with Silver–Russell syndrome (SRS), which compared with those with H19/IGF2:IG‐DMR epimutations has been suggested to have low frequency of body asymmetry, higher level of IGF1, and high frequency of cardiovascular anomalies and developmental delay [6].

We present two individuals, one child and one adult, with de novo *IGF2* variants, and review previously reported cases from the literature. Additionally, we demonstrate the use of methylation patterns from long‐read sequencing as a powerful tool to identify the allelic origin of de novo variants in the imprinted IGF2‐gene.

## 2 Methods

For detailed description of whole‐exome, whole‐genome and long‐read sequencing, please see Supporting Information.

### 2.1 Review

We included 18 cases from literature, which were non‐mosaic and had sufficient information to allow for comparison of clinical features [6, 7, 8, 9, 10, 11, 12, 13, 14, 15], as well as the two individuals from the present study in the review.

## 3 Results

### 3.1 Genetic Results

#### 3.1.1 Individual 1 (26‐Year‐Old Female)

Trio‐exome sequencing due to intellectual disability (ID) revealed a de novo heterozygous variant in IGF2: NM_000612.6:c.70G>C, p.(Ala24Pro).

Based on ACMG/AMP 2015 guidelines, the variant is classified as likely pathogenic.

Long‐read sequencing enabled haplotype phasing of the *IGF2* locus, assigning the c.70G>C variant to a single haplotype. Haploblock with transcript_id 1547.1 spanning chr11: 6221‐9616156 (9.6 Mb) (Figure 1). Comparison with parental haplotype identified this as the paternally inherited allele. Consistent with the established imprinting pattern, the paternal haplotype was methylated at *IGF2*:Ex9‐DMR (DMR2), supporting paternal‐specific methylation and expression of IGF2 (Figure S1).

**FIGURE 1:**
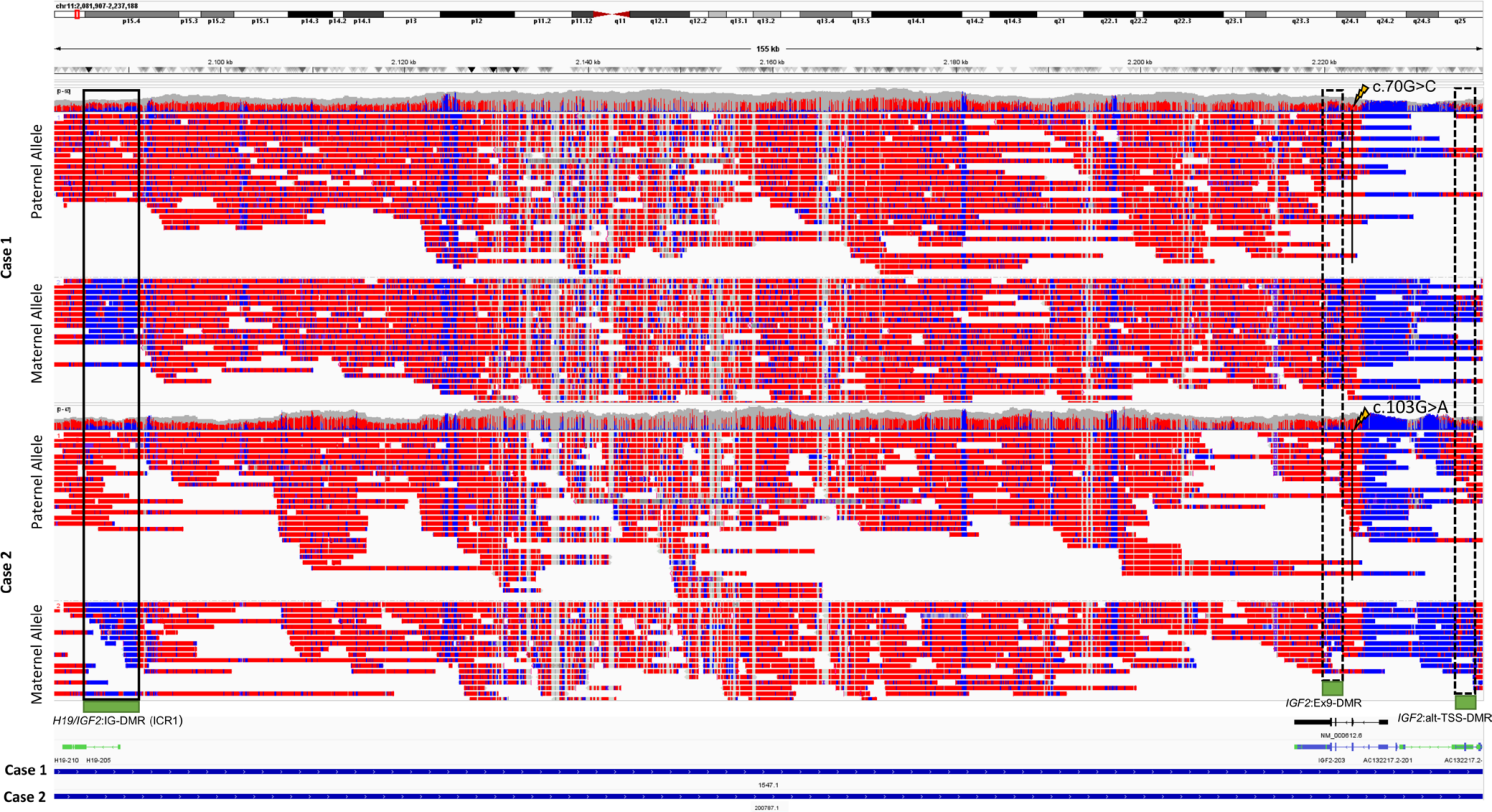
Long‐read haplotype phasing and allele‐specific methylation across the *IGF2*/*H19* locus. Long‐read sequencing generated phased haploblocks spanning the *IGF2/H19* imprinted region in both patients. In Case 1, the c.70G>C variant was assigned to a phased haploblock (transcript_id 1547.1) spanning chr11:6221–9616156 (9.6 Mb). In Case 2, the c.103G>A variant was assigned to a phased haploblock (transcript_id 200 787) spanning chr11:200787–2 711 017 (2.5 Mb). In both cases, the haploblocks encompassed the *H19/IGF2*:IG‐DMR (ICR1) together with the secondary *IGF2*:Ex9‐DMR and *IGF2*:alt‐TSS‐DMR. The concordant allele‐specific methylation observed across all three DMRs demonstrates that methylation at *IGF2*:Ex9‐DMR alone can serve as a marker of parental origin, enabling assignment of de novo IGF2 variants to the expressed paternal allele. [Colour figure can be viewed at wileyonlinelibrary.com]

#### 3.1.2 Individual 2 (5‐Year‐Old Female)

Trio‐genome sequencing (short read, Illumina) was performed due to poor growth, identifying a de novo heterozygous missense variant in *IGF2*: NM_000612.6:c.103G>A, p.(Gly35Arg).

Based on ACMG/AMP 2015 guidelines, the *IGF2* variant is classified as likely pathogenic.

Long‐read sequencing enabled haplotype phasing across the *IGF2* locus, placing the c.103G>A variant on a single phased haplotype, haploblock with transcript_id 200787 spanning chr11: 200787‐2711017 (2.5 Mb) (Figure 1). Comparison with parental haplotype assigned this haplotype to the paternal allele. In agreement with the established imprinting pattern, the paternal haplotype was methylated at *IGF2*:Ex9‐DMR (DMR2), supporting assignment of the variant to the expressed paternal allele (Figure S2).

### 3.2 Review

For detailed clinical descriptions of Individuals 1 and 2 please see Supporting Information and Figure 2.

**FIGURE 2:**
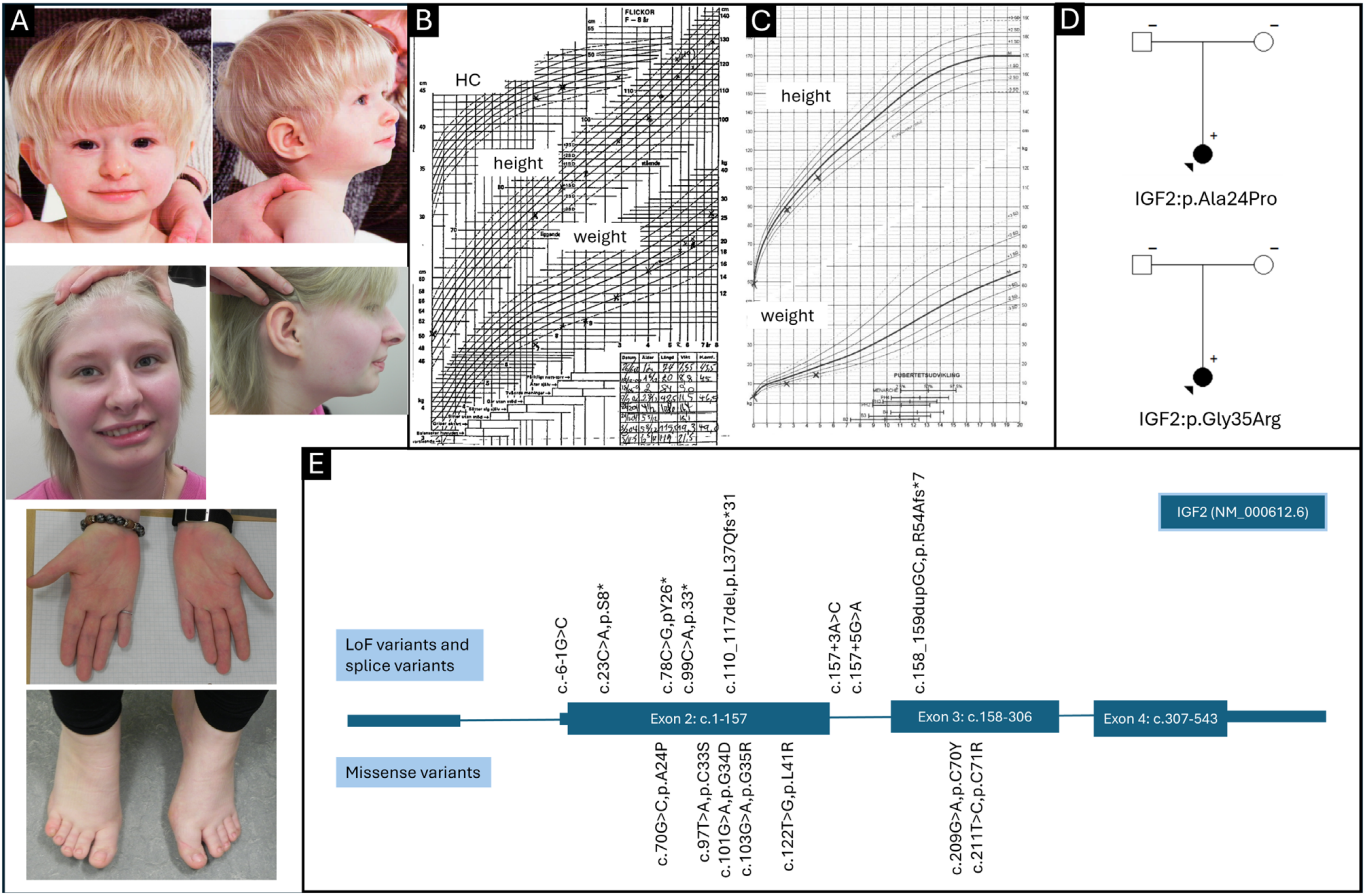
(A) Photos of Individual 1 at age 1.5 years old (left) and age 14 years (right). She was a slender infant with relative macrocephaly, protruding forehead, upward slanting palpebral fissures, low set ears, wide nasal bridge, low hanging columella, wide mouth, and retrognathia. At age 14 years, head circumference was normal. Fifth fingers were short, and feet were small. (B, C) Growth curves for Individual 1 and Individual 2, respectively. (D) Pedigrees. (E) The location of the sequence variants in IGF2 (NM_000602.6) reviewed in this paper (not to scale). The variants were selected as non‐mosaic cases with sufficient information to be part of the review [6, 7, 8, 9, 10, 11, 12, 14, 16]. A large deletion in 3′UTR, and a reciprocal translocation included in the review [13, 15] were not shown. [Colour figure can be viewed at wileyonlinelibrary.com]

Twenty individuals were included in the review (see Table 1). The reviewed cases included 10 males and 8 females with ages from 18 months to 31 years. In two families the variant was paternally inherited (one multigenerational family [7], and one with a reciprocal translocation [15]). Fifteen cases were sporadic, 14 of which had a confirmed de novo variant, with 11 shown to be located on the paternal allele. There were 17 unique variants: 5 nonsense or frameshift variants, 3 splice variants, 1 large deletion, 1 reciprocal translocation, and 7 missense variants (see Figure 2).

**TABLE 1:** Clinical features of non‐mosaic cases with *IGF2* variants from literature and the two individuals from the present study.

	Nucleotid change (NM_000612.6)	Protein change	Inheritance	Sex	Age at reporting	#SGA	#Relatve macrocephaly at birth	#Length < −2 SD at age 2 years	#Feeding difficulties &/or BMI ≤ −2 SDS at 24 months	#Protruding forehead 1–3 years	#Body asymmetry
Begemann et al. [7] (III:3)	c.23C>A	p.S8*	pat	M	> 20 years	X	X	X	X	X	
Begemann et al. [7] (III:4)	c.23C>A	p.S8*	pat	F	> 15.5 years	X	X	X	X	X	
Begemann et al. [7] (III:7)	c.23C>A	p.S8*	pat	M	> 13 years	X	X	X	X	X	
Begemann et al. [7] (IV:1)	c.23C>A	p.S8*	pat	F	18 months	X	X	NA	NA	X	
Habib et al. [8]	c.78C>G	p.Y26*	dn	M	16 years	X	X	X	X	X	
James et al. [9]	c.99C>A	p.C33*	dn, pat allele	NI	NI	X	NI	NI	NI	NI	
Yamoto et al. [14]	c.110_117delTGGTGGACinsAGGTAA	p.(Leu37Glnfs*31)	dn, pat allele	M	18 months	X	X	X	X	X	
Habib et al. [8]	c.158_159dupGC	p.(Arg54Alafs*7)	dn	F	NI	X	X	X	X	X	
Masunaga et al. (1) [6]	c.‐6‐1G>C		dn, pat allele	M	NI	X	X	X	X	X	
Poulton et al. [11]	c.157 + 3A>C		dn, pat allele	F	4 years	X	X	NI	NI	X	
Xia et al. [12]	c.157 + 5G>A		dn, pat allele	M	4 years	X	X	X	X	X	
Sun et al. [13]	c.*1768_*5188del3421 (3.4 kb deletion)		dn	NI	NI	X	NI	NI	NI	NI	
Murphy et al. [15]	t(1;11) (p36.22;p15.5)		pat	F	31 years	X	NI	NI	NI	NI	
Our study, Individual 1	c.70G>C	p.Ala24Pro	dn, pat allele	F	26 years	X	X	No	X	X	
Masunaga et al. (4) [6]	c.97 T>A	p.C33S	dn, pat allele	F	NI	X	X	X	X	X	
Liu et al. [10]	c.101G>A	p.G34D	dn, pat allele	M	13 years	X	X	X	X	NI	X
Our study, Individual 2	c.103G>A	p.Gly35Arg	dn, pat allele	F	5 years	X	X	No	X	X	
Loid et al. [16]	c.122 T>G	p.L41R	dn, pat allele	M	16 years	X	X	X	X	X	
Masunaga et al. (2) [6]	c.209G>A	p.C70Y	dn, pat allele	M	NI	X	X	X	X	X	
Masunaga et al. (3) [6]	c.211 T>C	p.C71R	dn or pat	M	NI	X	X	X	X	X	X
Total						20/20	17/17	13/15	15/15	16/16	2/20

	NH‐CSS	Growth hormone therapy	Adult height (SD)	Motor delay	Speech delay	ID, LD	Congenital heart defect	Hypoplastic placenta	Clinodactyly	Anomaly of extremity	Genital anomaly	Serum‐IGF1
Begemann et al. [7] (III:3)	5/6	X	−4 SD	X	X	ID				X		Normal
Begemann et al. [7] (III:4)	5/6	X	−2.7 SD	(X)	X	LD			X			Normal
Begemann et al. [7] (III:7)	5/6	X	−1.6 SD	No	No	No					X	Normal
Begemann et al. [7] (IV:1)	NA	NA	NA	X	X	NA				X		NI
Habib et al. [8]	5/6	X	NA	NI	NI	NI						+3.3 SD
James et al. [9]	NA	NI	NA	X	X	NI	VSD			X		NI
Yamoto et al. [14]	5/6	No	NA	X	X	NA		X	X	X	X	+6.1 SD
Habib et al. [8]	5/6	X	NA	NI	NI	NI						+5.4 SD
Masunaga et al. (1) [6]	5/6	NI	NA	X	X	ID	X	X	X			(High)
Poulton et al. [11]	SRS	X	NA	X	X	NA	VSD		X			Normal
Xia et al. [12]	5/6	NI	NA	X	X	NA		X	X			NI
Sun et al. [13]	SRS	NI	NA	X	X	NI					X	NI
Murphy et al. [15]	NI	NI	−3 SD	No	No	No						Normal
Our study, Individual 1	4/6	No	+0.1 SD	X	X	ID						Normal
Masunaga et al. (4) [6]	5/6	X	NA	No	No	NI			X	X		(High)
Liu et al. [10]	5/6	X	NA	No	No	No	ASD		X		X	−2 SD
Our study, Individual 2	4/6	No	NA	No	X	NA				X		Normal
Loid et al. [16]	5/6	X	NA	No	No	NI		X	X		X	High to normal
Masunaga et al. (2) [6]	5/6	X	NA	X	X	NI		X	X		X	(High)
Masunaga et al. (3) [6]	6/6	X	NA	X	X	NI		X	X		X	(High)
Total		11/13		12/18	12/18	4/6						

*Note:* In the column of NH‐CSS, SRS (Silver–Russell Syndrome) indicates individuals from studies of SRS, where the NH‐CSS score was not given for the individual. In the column of serum‐IGF1, (High) indicates that serum‐IGF1 was described as “values tended to be high,” but data was not given for each individual.

Abbreviations: #: features of the NH‐CSS (Netchine‐Harbison Clinical Scoring System); ID: intellectual disability; LD: learning disability; NA: not assessed; NI: no information; pat = paternal.

The Netchine‐Harbison Clinical Scoring System (NH‐CSS) [17, 18, 19] was applied in 15 individuals, of which 1 scored 6/6 points, 12 scored 5/6, and 2 scored 4/6 points, thus fulfilling the criteria for SRS. All were born small for gestational age, and in six cases, a hypoplastic placenta was described. Body asymmetry was only noted in two individuals. While most individuals (13/15) had length below −2 SD at age 2 years, the two individuals from our study had length in the normal range, at birth and at age 2 years. Of five reported adults, three had short stature, and two had height in the normal range.

Delayed motor and speech development was noted in two thirds (12/18). Information about cognitive function was only available for six individuals, of whom three had ID, one had learning disability (LD), and two had normal cognition.

Other features were genital anomaly (mostly under‐masculinized male genitalia or hypospadia), anomalies of hands or feet ranging from clinodactyly and small hands/feet to SFHM (Split hand foot malformation) which was seen in three individuals [6, 7, 14] and congenital heart malformation which was reported in four individuals (primarily ASD and VSD).

For those individuals where serum IGF‐1 levels were given, three had values > +2 SD, and four had values in the normal range.

## 4 Discussion

In the review of clinical features in 20 individuals with *IGF2* variants, we found the most frequent features to be those of SRS with low birth weight, relative macrocephaly at birth, short stature and feeding difficulties, and protruding forehead in infancy, while body asymmetry was rarely seen (NH‐CSS). Other recurrent features were delayed speech and motor development, under‐masculinized male genitalia, and anomalies of hands and feet ranging from clinodactyly and small hands/feet to SFHM in a few individuals. A congenital heart anomaly was reported in some individuals.

It is interesting that while most cases in literature are seen with faltered growth affecting length as well as weight, the two individuals from our study had low weight but height in the normal range from birth and on. Neither Individual 1 nor Individual 2 received growth hormone treatment. Growth was only described in five adults, of whom two had height in the normal range, and three had short stature. Individual 1 from this study was the one with the most convincing catchup of growth. Her thriving increased spontaneously from approximately age 3 years, and as an adult she is mildly overweight with a height of +0.1 SD. The variant of this patient, p.Ala24Pro, is located as the earliest positioned of all missense variants included in the review, and not included in the small cluster of missense variants affecting aminoacids 33–35. This large difference in pattern of faltering growth might indicate existence of a genotype–phenotype correlation still to be explored.

While most individuals had delayed motor and speech development, information on cognition was only available for six, of whom three had either ID or LD. While a diagnosis of ID cannot be assessed in young children, the lack of information on cognition in the remaining cases can either be caused by cognition not being a focus of investigation or it not being mentioned if cognition was normal. Whether ID is a frequent feature of IGF2 is thus uncertain at this point.

High pretreatment serum IGF‐1 has been suggested to be a feature of *IGF2*‐related SRS [6]. We find that while some individuals clearly had high serum IGF‐1, others had clearly normal values. High serum IGF1‐1 is thus not a feature of all individuals with SRS caused by *IGF2* variants.

Traditionally, determining the parental origin of de novo variants in imprinted genes has required parental samples and informative SNP‐based phasing. However, long‐read sequencing enables simultaneous phasing of genetic variants and allele‐specific methylation, allowing parental origin to be assigned directly from the proband without trio analysis [20, 21]. We were able to confirm that the de novo variants in both our *IGF2* cases were present on the hypermethylated paternal allele, consistent with the established imprinting pattern at the *IGF2* locus. Of note, although the secondary *IGF2* DMRs (*IGF2*:Ex9‐DMR (DMR2) and *IGF2*:alt‐TSS‐DMR (DMR0)) exhibit partial and inter‐individually variable methylation, their allele‐specific methylation is coordinated by the primary imprinting control region, *H19*/*IGF2*:IG‐DMR (ICR1), which displays a stable parent‐of‐origin–specific differential methylation [2, 3, 21, 22, 23]. Our findings demonstrate the clinical applicability of this approach for *IGF2*, where methylation at *IGF2*:Ex9‐DMR reliably identified the disease‐causing variant as residing on the expressed paternal allele.

In conclusion, we report two individuals with de novo variants in the imprinted IGF2 gene and review published cases. We find that there are several features differing between individuals, including the pattern of growth faltering, ID, and S‐IGF1 levels. We suggest that a genotype–phenotype correlation most likely exists explaining these differences, which could be a focus of future studies. We demonstrate the use of methylation patterns from long‐read sequencing as a powerful tool to identify the allelic origin of de novo variants in the imprinted *IGF2* gene.

## Author Contributions

All authors have contributed to the writing and approval of the manuscript.

## Consent

A written informed consent was obtained from all individuals described in this report including the publication of photographs.

## Conflicts of Interest

The authors declare no conflicts of interest.

## Supporting information


**Figure S1:** Phasing of methylome and genome variation of *IGF2* in Individual 1.
**Figure S2:** Phasing of methylome and genome variation of IGF2 in Individual 2.

## Data Availability

The data that support the findings of this study are available on request from the corresponding author. The data are not publicly available due to privacy or ethical restrictions.
