# Supplementary material for: Variants in the Imprinted IGF2 Gene: A Review and Phasing of De Novo Variants Using Long‐Read Sequencing
Source: Clin Genet. 2026 Sep 1;110(6):748–55. doi: 10.1111/cge.70239 (PMC13728960; doi:10.1111/cge.70239)
Supplement: Supplementary file 1 [file CGE-110-748-s001.docx]

**Methods:**

**Illumina whole-exome and whole-genome sequencing**

DNA was extracted from peripheral blood samples using Tecan Freedom EVO® (Tecan, Switzerland). DNA libraries were quantified using the Qubit dsDNA BR Assay Kit (Thermo Fisher Scientific, MA, USA). For WES libraries, we used the NimbleGen SeqCap EZ Library SR kit (Roche, Switzerland). For WGS libraries, we used Illumina DNA PCR-Free Library Prep (Illumina, CA, USA). In both cases, the prepared libraries were paired-end sequenced on the NovaSeq 6000 platform (Illumina, CA, USA) following the manufacturer's protocol and processed using the Illumina DRAGEN Bio-IT platform (Illumina, CA, USA). Variant filtering was carried out using VarSeq 2.6.2 (Golden Helix, MT, USA).

**Nanopore long-read genome sequencing**

Genomic DNA was extracted using high molecular weight DNA extraction kit for cells and blood (NEB Monarch #T3050) according to the protocol provided by the manufactures. DNA libraries were prepared using ligation sequencing gDNA kit SQK-LSK112 and SQK-LSK114 (Oxford Nanopore Technology (ONT)) and sequenced as whole genomes on R10.4.1 flow cells using the PromethION P24 instrument (ONT). Basecalling was performed with the SUP model including modified 5mC detection in Dorado v0.8.3. Reads were processed by the human variation workflow v2.5.1 provided by ONTs epi2me labs and visualized in IGV genome browser, analysis performed with both hg38 and T2T-CHM13, visualization and phasing were performed using the T2T-CHM13 assembly, where alignment is unambiguous.

**Detailed clinical description of individuals 1 and 2.**

**Individual 1**

This 26-years-old female was born at gestational age (GA) 40+6 weeks, with birth weight (BW) 2275 g (-3 SDS), and birth length (BL) 50 cm (median). Parents were healthy, however the mother had epilepsy from age 5-14 years.

At age 7 months, she was admitted to hospital with poor thriving, length and head circumference were in normal range, but weight at -4SD. She was described with triangular face, pointed nose with broad nasal ridge, bilateral four finger crease, and overlapping second toes. Poor thriving was prominent until age three years, where she was described with a large appetite, and weight increased to normal range. At age 18 years, H was 168 cm (just below median), BMI 26.6 (overweight), and HC 54 cm (median). Puberty was normal. She has always had a tendency for headaches. Plasma-IGF-1 at age 2 years was normal (7 m: 103 ng/ml (+1SD), 2 y: 127 ng/ml (+1SD)).

Psychomotor development was delayed. She walked at age 2-2½ years. Speech was delayed, and she spoke only few words at age 18 months. She was described as socially challenged. Cognitive testing (WPPSI) at age 4 years indicated a developmental age of 2-2.5 years. Total IQ was 40 at age 15 years (WISC-III), and 54 at age 17 years (WAIS-IV), corresponding to mild to moderate intellectual disability (ID). At age 5 years, she developed epilepsy with generalized seizures, which was successfully controlled by medical treatment (valproate). She has poor balance and dysarthria.

Genetic testing with normal result: 46,XX, FISH for Prader Willi syndrome, multisubtelomer FISH, FISH for selected microdeletion syndromes, exons 32-34 in SRCAP; Chromosomal microarray.

**Individual 2**

This 5-years-old female showed signs of intrauterine growth restriction (IUGR) (-10%) at GA 33+1 despite normal function of the placenta. The mother had hypothyroidism and was treated with Levothyroxine Sodium tablets during pregnancy. She was born at GA 40+3 by caesarian section due to prolonged labors. BW was 2845 g (-2 SDS), BL 49 cm (-1 SDS), and OFC 35 cm (median). Placental weight was 560 g (normal range).

At age 2 years and 6 months, height was 89 cm (-1 SDS), weight 10.2 kg (-3 SDS), and OFC 46.5 cm (-1.5 SDS), and at 4 years and 8 months, height was 105 cm (-1 SDS), weight 14.5 kg (-2 SDS) and OFC 48.5 cm (-1.5 SDS) according to WHO growth standards. The parents were of Sri Lankan descent, paternal height was 173 cm, and maternal height 169 cm. S-IGF was in low normal range (5 y: IGF1 (81 µg/L)).

She had frequent middle ear infections in infancy, and by age 1 year and 3 months she was treated for varicella zoster encephalitis.

Motor milestones were normal, and she walked at age 14 months. Speech development was mildly delayed, as she spoke her first word at 19 months. By age 5 years, her psychomotor development and her social skills are normal.

She has mild retrognathia, and the second toes overlaps the first toes on both sides. She has severe myopia (-15 diopter, D) and pseudoproptosis, and congenital stationary night blindness is suspected.

**Supplementary figures**

**Supplementary Figure 1.** Phasing of methylome and genome variation of *IGF2* in Individual 1(Case 1). Visualization of nanopore long-read sequencing in IGV. The long reads spanning the *IGF2*:Ex9-DMR, the pathogenic 70G>C variant (marked by the thunderbolt sign) and the informative SNP (marked by a star) and 6 informative SNPs on the materlan allele (marked by blue box). Methylation is marked in red; unmethylated CpGs are represented in blue. Phasing of the two alleles, using both methylation(upper picture) and trio genome with SNP positions (lower picture) showing that the *de novo* *IGF2* variant is located on the paternally inherited allele.

**Supplementary Figure 2.** Phasing of methylome and genome variation of IGF2 in Individual 2. Visualization of nanopore long-read sequencing in IGV. The long reads spanning the *IGF2*:Ex9-DMR, the pathogenic c.103G>A variant (marked by the thunderbolt sign) and the informative SNP (marked by a star). Methylation is marked in red; unmethylated CpGs are represented in blue. Phasing of the two alleles, using both methylation(upper picture) and trio genome with SNP positions (lower picture) showing that the *de novo* *IGF2* variant is located on the paternally inherited allele.
